# Effect of Digoxin Therapy on Mortality in Patients With Atrial Fibrillation: An Updated Meta-Analysis

**DOI:** 10.3389/fcvm.2021.731135

**Published:** 2021-10-01

**Authors:** Xiaoxu Wang, Yi Luo, Dan Xu, Kun Zhao

**Affiliations:** ^1^Department of Cardiovascular Diseases, The First Branch, The First Affiliated Hospital of Chongqing Medical University, Chongqing, China; ^2^Department of Sports Medicine, Zhejiang University School of Medicine, Hangzhou, China

**Keywords:** digoxin, atrial fibrillation, heart failure, mortality, readmission

## Abstract

**Background:** Whether digoxin is associated with increased mortality in atrial fibrillation (AF) remains controversial. We aimed to assess the risk of mortality and clinical effects of digoxin use in patients with AF.

**Methods:** PubMed, Embase, and the Cochrane library were systematically searched to identify eligible studies comparing all-cause mortality of patients with AF taking digoxin with those not taking digoxin, and the length of follow-up was at least 6 months. Hazard ratios (HRs) with 95% confidence intervals (CIs) were extracted and pooled.

**Results:** A total of 29 studies with 621,478 patients were included. Digoxin use was associated with an increased risk of all-cause mortality in all patients with AF (HR 1.17, 95% CI 1.13–1.22, *P* < 0.001), especially in patients without HF (HR 1.28, 95% CI 1.11–1.47, *P* < 0.001). There was no significant association between digoxin and mortality in patients with AF and HF (HR 1.06, 95% CI 0.99–1.14, *P* = 0.110). In all patients with AF, regardless of concomitant HF, digoxin use was associated with an increased risk of sudden cardiac death (SCD) (HR 1.40, 95% CI 1.23–1.60, *P* < 0.001) and cardiovascular (CV) mortality (HR 1.27, 95% CI 1.08–1.50, *P* < 0.001), and digoxin use had no significant association with all-cause hospitalization (HR 1.13, 95% CI 0.92–1.39, *P* = 0.230).

**Conclusion:** We conclude that digoxin use is associated with an increased risk of all-cause mortality, CV mortality, and SCD, and it does not reduce readmission for AF, regardless of concomitant HF. Digoxin may have a neutral effect on all-cause mortality in patients with AF with concomitant HF.

**Systematic Review Registration:** https://www.crd.york.ac.ukPROSPERO.

## Introduction

Atrial fibrillation (AF) is one of the most common types of arrhythmias worldwide. The currently estimated prevalence of AF in adults is between 2 and 4% (1). The prevalence of AF could continue to rise, owing to aging of the general population (2) and intensified screening for undiagnosed AF using various detection devices (3). Atrial fibrillation is associated with an increased risk of stroke and transient ischemic attack (4). Recently, a large meta-analysis showed a high prevalence of heart failure (HF) ranging from 6.42 to 55.78% in cohorts with AF (5). Patients with AF often have co-existing HF, which worsens prognosis (2). Therefore, AF is associated with substantial morbidity and mortality, posing a significant burden to patients. Heart rate control is an integral part of AF management. Digoxin is a cardiac glycoside derived from *Digitalis lanata*. Since the 1960s, digoxin has played a major role as a therapeutic agent for heart rate control in patients with AF or HF (6). Digoxin exerts chronotropic effects via parasympathetic activation and inotropic effects through inhibition of the sodium–potassium ATPase, promoting activity of the sodium–calcium exchanger and increasing intracellular calcium concentration, which subsequently increases contractility (6, 7). Owing to its negative chronotropic activity, digoxin is still commonly used for heart rate control in patients with AF or HF, particularly in those who do not achieve their heart rate target or who are unable to tolerate β-blocker therapy. During recent years, data on the safety of digoxin treatment in patients with AF continue to emerge. Many observational studies indicate that digoxin has potentially harmful effects in patients with AF (8–10). Moreover, several meta-analyses suggest that digoxin is associated with an increased risk of mortality in patients with AF (11–13). However, neutral effects on mortality in patients with AF receiving digoxin therapy were also reported (14, 15). Even the most recent meta-analysis showed no evidence of a difference in all-cause mortality in patients with AF receiving digoxin therapy compared with those receiving a control intervention (16). Moreover, 2020 European Society of Cardiology guidelines for AF management recommend digoxin in patients with HF with reduced ejection fraction as a class I indication (level B) (3). A series of new and conflicting studies have been published, and it remains controversial as to whether digoxin is associated with increased mortality in patients with AF. It also remains unclear whether digoxin use is associated with reduced hospitalization in patients with AF (15, 17). Furthermore, few meta-analyses have focused on serious adverse events, such as systemic embolic event (SEE), myocardial infarction (MI), cardiovascular (CV) mortality, and sudden cardiac death (SCD). Therefore, we performed a meta-analysis to evaluate the risk of mortality and readmission with digoxin in patients with AF with or without HF. We also compared the risk of serious adverse events in patients taking digoxin with those not taking digoxin.

## Methods

This meta-analysis was performed in accordance with Preferred Reporting Items for Systematic Reviews and Meta-Analyses guidelines (PRISMA) (18). The project was prospectively registered in the PROSPERO database (CRD42020222258). Literature searches of PubMed, Embase, and the Cochrane Library were performed to identify and retrieve all potentially relevant articles related to this topic. The searches were performed utilizing the following keywords: “digoxin” OR “digitalis” OR “digitoxin” AND “atrial fibrillation” until September 2020. See the attachment for more details on the search strategy. The search was limited to human research, the study design was limited to observational studies or retrospective analyses of randomized controlled trials (RCTs), and the language was restricted to English. A manual search was also performed by examining the reference lists of included studies.

Two independent investigators (WXX and LY) screened the citations through title and abstract. Studies were included if: (1) digoxin compared with no digoxin or other heart rate control treatment in patients with AF; (2) hazard ratios (HRs) with 95% confidence intervals (CIs) for outcomes associated with digoxin treatment were reported; (3) the length of follow-up was at least 6 months; (4) all-cause mortality was the endpoint. The exclusion criteria were: studies that did not provide comparative outcomes, studies that did not report the association between digoxin use and mortality, studies were not published as full text articles and data were derived from the same study.

Two investigators (WXX and LY) independently reviewed the full manuscripts of included studies and extracted information into an electronic database, including author names, year of publication, study design, number of participants, follow-up duration, outcomes, unadjusted HRs or HRs adjusted by statistical models (propensity score matched model or non-propensity score matched model). Any discrepancies between the two investigators regarding data extraction were resolved by consensus after discussion with a third investigator (XD).

Two investigators (WXX and LY) independently assessed the methodological quality of each study using the Newcastle–Ottawa scale. The Newcastle–Ottawa scale comprises three parts: patient selection, study comparability, and outcome assessment. The Newcastle–Ottawa scale assigns a maximum of 4 points for selection, 2 points for comparability, and 3 points for outcome. Therefore, a score of 9 points indicates the highest quality, 6–8 points indicates medium quality, and <6 points indicates low quality. Any discrepancies were resolved by discussion with a third investigator (XD).

A traditional meta-analysis was performed on studies reporting outcomes associated with digoxin use in patients with AF. The primary outcome was all-cause mortality, and the secondary outcomes were all-cause hospitalization and serious adverse events, including SEE /stroke, MI, CV mortality, non-CV mortality, and SCD. Stata software (version 16.0; Stata Corp. LP., College Station, Texas) was used to pool the data and perform the statistical analysis. HRs and CIs were transformed logarithmically, and the inverse variance method was used to achieve a weighted estimate of the combined overall effect. Statistical heterogeneity was assessed by visual inspection of forest plots and by calculating the *I*^2^ statistic. Significant heterogeneity was considered present at a 5% level of significance (for the Q-test) and an *I*^2^-value of > 50%. The primary outcome analysis (Q-test: *P* < 0.001; *I*^2^ = 85.82%) and most subgroup analyses exhibited significant heterogeneity; therefore, we adopted the random-effects model. Subgroup analyses of the primary outcome were conducted according to cardiac function (with HF vs. without HF), digoxin exposure (baseline digoxin use when enrolled vs. digoxin initiation during follow-up), age (>80 vs. <80 years), and statistical methods (unadjusted HRs or HRs adjusted by propensity score matched model or non-propensity score matched model). Publication bias was assessed by inspecting funnel plots in which the natural log of the HR was plotted against its standard error and further tested by Begg's-test and Egger's-test. *P*-values were two-sided, and a *P*-value of < 0.05 was considered statistically significant.

## Results

**Figure 1:**
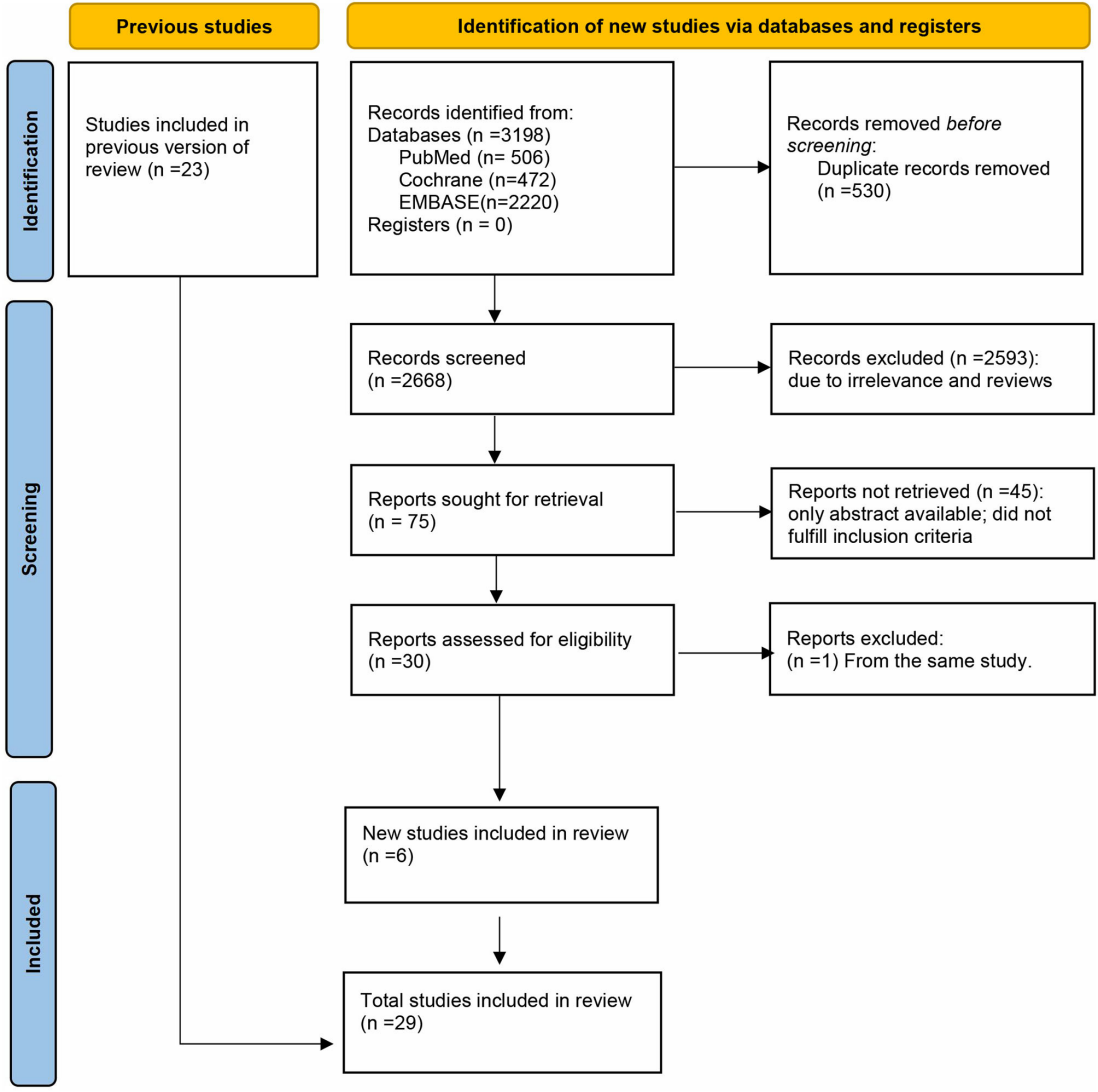
PRISMA flow diagram of the study selection.

A total of 3,198 manuscripts were initially identified. After removing duplicates, 2,668 studies remained. After screening titles and abstracts, 75 studies remained. After full review of the 75 manuscripts, we found that a study by Gheorghiade et al. (19). and a study by Whitbeck et al. (20) were both based on the same original trial database (Atrial Fibrillation Follow-up Investigation of Rhythm Management, AFFIRM), thus we only included the former because it had a rigorous analytic methodology and provided data on hospitalization in patients with AF using digoxin compared with those without digoxin. We finally included 29 studies (8–10, 14, 15, 17, 19, 21–42), in which there were 6 new studies (14, 15, 38, 40–42) different from previous version of systematic review (Figure 1). And a total of 621,478 patients were included in the meta-analysis. On the basis of the Newcastle–Ottawa scale, two studies were of high quality (24, 32), while 27 studies were of medium quality.

The characteristics of studies are summarized in Table 1. There were four studies (15, 23, 38, 42) only included patients with AF with concomitant HF, while one study (32) only included patients with AF without concomitant HF. The remaining 24 studies included patients with AF regardless of concomitant HF. Twenty-six studies reported digoxin therapy in patients with AF at the time of registry enrollment, while the other five studies provided data for digoxin initiation in patients with AF during follow-up. Data of eight studies were based on retrospective analyses of RCTs, and the remaining 21 studies were observational cohort studies.

**Table 1:** Characteristics of the included studies.

**Study, Year (Reference)**	**Country**	**Study design**	**Heart function**	**Sample size**	**Digoxin exposure**	**Follow-up (Y)**	**Age (Y)**	**Outcomes**	**Statistical models**	**NOS**	**Main variables adjusted by statistical models**
Hallberg2007 (21)	Swedish	Prospective registry study	With or without HF^*^	60,764	Baseline use	1	76	All-cause mortality	NPSM, CR	7	Age, gender, smoking, history of DM or hypertension or HF, pacemaker, medication (ACEi, betablockers, antiplatelet drugs, lipid-lowering drugs, anticoagulation, diuretic)
Gjesdal2008 (22)	USA	Retrospective analysis of RCT	With or without HF	7,329	Baseline use	1.5	71	All-cause mortality; SSE	NPSM, CPH	6	Age, gender, BMI, BP, smoking, history of DM or hypertension or CAD, prior stroke or TIA, prior SEE, years since first AF diagnosis, LV dysfunction, medication (aspirin, betablockers)
Fauchier2009 (23)	France	Retrospective cohort study	With HF	1,269	Baseline use	2.41	74	All-cause mortality	NPSM, CPH	8	Age, gender, history of DM or hypertension or CAD or valvular disease or PVD or renal insufficiency or pulmonary disease, prior stroke/TIA or MI or PCI or CABG, Permanent AF, CHADS2-score, LVEF, pacemaker, medication (ACEi, diuretic, anticoagulation, antiarrhythmics)
Friberg2009 (24)	Sweden	Prospective registry study	With or without HF	2,824	Baseline use	4.6	78	All-cause mortality; HF hospitalization; stroke; MI	PSM, CR^*^	9	Age, gender, history of DM or hypertension or MI or valvular disease or PVD or renal insufficiency or pulmonary disease, prior stroke/TIA, permanent AF, LVEF, CHADS2-score, pacemaker, medication (ACEi/ARB, aspirin, betablockers, warfarin)
Gheorghiade 2013 (19)	USA	Retrospective analysis of RCT	With or without HF	1,756	Initial use	3.5	70	All-cause mortality; all-cause hospitalization; CV mortality; non-CV mortality	PSM	8	Age, gender, history of DM or hypertension or CAD or valvular disease or PVD or renal disease or pulmonary disease or cerebrovascular events, prior MI or CABG, LVEF, pacemaker, medication (ACEi, aspirin, betablockers, warfarin)
Shah2014 (29)	Canada	Retrospective cohort study	With or without HF^*^	74,234	Baseline use	3.0-4.2	80.3	All-cause mortality	PSM	8	Age, gender, history of DM or hypertension or CAD or MI or valvular disease or renal insufficiency, medication (ACEi/ARB, betablocker, diuretic, warfarin)
Mulder2014 (27)	Netherlands	Retrospective analysis of RCT	With or without HF	608	Baseline use	2.9	68	All-cause mortality; CV hospitalization; CV mortality; non-CV mortality; stroke	NPSM, CPH	6	Age, gender, history of HF, total duration of AF, NYHA class, creatinine, N-Terminal brain natriuretic peptide, QRS duration, medication (ACEi/ARB, betablocker, diuretics)
Rodriguez-Manero2014 (28)	Spain	Prospective registry study	With or without HF	777	Baseline use	2.9	74.9	All-cause mortality; all-cause hospitalization	NPSM, CPH	7	Age, gender, BMI, smoking, history of DM or hypertension or CAD or HF or dyslipidemia or renal insufficiency, prior stroke, LVEF, medication (betablocker, CCB, antiarrhythmic drugs)
Chao2014 (25)	Taiwan, China	Retrospective cohort study	With or without HF^*^	4,781	Baseline use	4.32	67.8	All-cause mortality; stroke	NPSM, CR	7	Age, history of hypertension or DM or HF or COPD, CHA2DS2-score, medication (beta-blockers, CCB and ACEi/ARB)
Gamst2014 (26)	Danish	Prospective registry study	With or without HF	8,880	Baseline use	1	80	All-cause mortality	NPSM, CR	7	Age, gender, obesity, history of hypertension or DM or HF or MI or PVD or pulmonary diseases or valve disease or renal disease, prior TIA or cerebrovascular disease, medication (betablocker, Aspirin, CCB, statins, warfarin)
Turakhia2014 (30)	USA	Retrospective cohort study	With or without HF	122,465	Baseline use	2.88	72.1	All-cause mortality	PSM, CR^*^	8	Age, gender, history of HF or hypertension or DM, prior stroke, glomerular filtration rate, CHADS2-score, medication (diuretics, statins, warfarin, beta-blockers, ACE inhibitors/ARB, antiarrhythmic drug)
Okin2015 (33)	USA	Retrospective analysis of RCT	With or without HF	937	Baseline use	4.7	70	All-cause mortality; CV mortality; SCD	NPSM, CR	6	Age, history of DM or CAD or HF or stroke, QRS duration, heart rate, pulse pressure, serum glucose, creatinine, high-density lipoprotein cholesterol
Washam2015 (9)	Multicenter	Retrospective analysis of RCT	With or without HF	14,171	Baseline use	1.94	73	All-cause mortality; all-cause hospitalization; SSE; MI	NPSM, CPH	7	Age, gender, BMI, BP, heart rate, smoking, history of HF or hypertension or DM or COPD or vascular disease, AF type, prior stroke or TIA, creatinine clearance, medication (ACEi/ARB, betablocker, Aspirin, CCB, statins, warfarin, antiarrhythmics)
Al-Zakwani2015 (35)	Middle East	Prospective registry study	With or without HF^*^	1,962	Baseline use	1	56	All-cause mortality	NPSM, LR	8	Age, gender, BMI, history of hypertension or DM or CAD or COPD or PVD, prior stroke/ TIA, LV systolic dysfunction, creatinine, CHADS2-score, AF type, medications (diuretic, betablocker, statin, aspirin, warfarin)
Allen2015 (31)	USA	Prospective registry study	With or without HF^*^	9,619	Baseline use or initial use	1.83	75	All-cause mortality; all-cause hospitalization; CV hospitalization	NPSM, LR	7	Gender, BP, heart rate, history of DM or MI or hyperthyroidism or COPD, LVEF, renal function, AF type, NYHA class, medication (antiarrhythmic drug)
Freeman2015 (32)	USA	Retrospective cohort study	Without HF	14,787	Initial use	1.17	71.7	All-cause mortality; all-cause hospitalization	PSM	9	Age, gender, BMI, history of hypertension or DM or CAD or PVD or valvular disease, renal function, prior MI or PCI or CABG, prior stroke/TIA, medication (ACEi/ARB, betablocker, diuretics, CCB, statins, warfarin)
Pastori2015 (34)	Italy	Prospective registry study	With or without HF	815	Baseline use	2.77	73	All-cause mortality; CV mortality	PSM, CR^*^	7	Age, gender, history of hypertension or MI or CAD or DM or stroke or TIA, medication (antiplatelet drugs, beta blockers, verapamil and amiodarone)
Chao2015 (8)	Taiwan, China	Retrospective cohort study	With or without HF	207,576	Baseline use	4.9	70	All-cause mortality	PSM, CR^*^	8	Age, gender, history of DM or hypertension or HF or dyslipidemia or ventricular arrhythmias or chronic kidney disease or COPD, prior stroke/ TIA, medications (ACEi/ARB, aspirin, statins, warfarin)
Adedin sewo2017 (17)	USA	Retrospective cohort study	With or without HF	11,297	Baseline use	1	unclear	All-cause mortality; all-cause hospitalization	NPSM, CPH	6	Age, gender, history of valvular disease or dyslipidemia or chronic kidney disease or thyroid disease or gastrointestinal bleed, prior MI or PCI or CABG, ablation procedure, CHADS2, medications (warfarin, amiodarone, betablocker)
Eisen2017 (36)	USA	Retrospective analysis of RCT	With or without HF^*^	21,105	Baseline use	2.8	72	All-cause mortality; HF hospitalization; CV mortality; non-CV mortality; SCD; SSE; MI	PSM, CPH^*^	7	Age, gender, smoking, history of HF or hypertension or DM or CAD or MI or PVD or valvular disease or chronic obstructive pulmonary disease, prior stroke/ TIA, LVEF, creatinine, AF type, medications (warfarin, antiarrhythmics, ACEi/ARB, diuretic)
Wu2017 (37)	China	Prospective registry study	With or without HF^*^	1,991	Baseline use	1	68.5	All-cause mortality; CV mortality; SCD	NPSM, CPH	7	Age, gender, BMI, BP, heart rate, smoking, history of HF or hypertension or DM or MI or CAD or valvular disease or COPD, prior stroke or TIA, LVEF, AF type, medications (warfarin, aspirin, statin, betablocker, ACEi/ARB, CCB)
Lopes2018 (10)	USA	Retrospective analysis of RCT	With or without HF^*^	17,897	Baseline use or initial use	0.5	69	All-cause mortality; HF hospitalization; CV mortality; SCD; non-CV mortality	NPSM, CR	7	Age, gender, history of hypertension or DM or CAD or renal disease, prior MI or PCI, prior stroke/ TIA, NYHA class, LVEF, creatinine, AF type, medications (aspirin, diuretic, betablocker, ACEi/ARB)
Karthi keyan2018 (41)	Multicenter	Retrospective cohort study	With or without HF^*^	1,058	Baseline use	2	32.3	All-cause mortality; all-cause hospitalization	NPSM, LR	7	Age, gender, BMI, history of valvular disease or HF, prior stroke, NYHA class, LVEF
Gonzalez-Loyola2018 (38)	Spain	Retrospective cohort study	With HF	4,908	Baseline use	2.24	80	All-cause mortality	NPSM, CPH	8	Age, gender, smoking, history of CAD or HF or stroke or chronic kidney disease or COPD, LVEF, medications (diuretics, ACEi/ARB)
Yu2018 (39)	Korea	Retrospective cohort study	With or without HF^*^	7,034	Baseline use	4.5	63.6	All-cause mortality	PSM, CR^*^	8	Age, gender, history of hypertension or DM or HF or dyslipidemia or chronic kidney disease, prior stroke or TIA
Kodani2019 (14)	Japan	Prospective registry study	With or without HF	7,018	Baseline use	2	69.7	All-cause mortality; CV mortality; non-CV mortality; SCD	PSM, CPH^*^	8	Age, gender, heart rate, BMI, AF type, history of HF or hypertension or DM or CAD or cardiomyopathy, prior stroke/TIA, creatinine clearance, medications (warfarin, antiplatelet drugs, betablocker, CCB, antiarrhythmics)
Gao2019 (40)	China	Prospective registry study	With or without HF	10,472	Baseline use	2.96	69.66	All-cause mortality; CV hospitalization; CV mortality	NPSM, CPH	8	Age, gender, BMI, BP, heart rate, history of HF or DM or CAD, glomerular filtration rate, AF type, stroke/TIA, medications (anticoagulation, ACEi/ARB, antiarrhythmics, betablocker, CCB)
Elayi2020 (42)	USA	Retrospective analysis of RCT	With HF	1,376	Baseline use	3.08	unclear	All-cause mortality; CV hospitalization; CV mortality; non-CV mortality	NPSM, CR	7	Age, gender, BMI, heart rate, history of hypertension or DM or CAD or COPD, prior MI or CABG, prior stroke/ TIA, NYHA class, LVEF, glomerular filtration rate, AF type, medications (anticoagulation, betablocker, ACEi/ARB)
Singh2020 (15)	USA	Prospective registry study	With HF	1,768	Initial use	4	79	All-cause mortality; all-cause hospitalization; HF hospitalization	PSM	8	Age, gender, smoking, BP, heart rate, history of hypertension or DM or CAD or COPD or PAD, prior MI or CABG or PCI, prior stroke/ TIA, LVEF, creatinine, medications (aspirin, warfarin, betablocker, diuretics, ACEi/ARB, CCB)

*HF, heart failure; Y, year; RCT, randomized controlled trial; NOS, Newcastle–Ottawa scale; CV, Cardiovascular; MI, Myocardial infarction; SSE, Systemic Embolic Event; SCD, sudden cardiac death. PSM, Propensity Score-Matched; NPSM, non-Propensity Score-Matched; CR, Cox Regression; CPH, Cox Proportional Hazards; LR, Logistic regression; BMI, body mass index; BP,blood pressure; DM,diabetes mellitus; CAD, coronary artery disease; COPD, chronic obstructive pulmonary disease; PVD,peripheral vascular disease; MI, myocardial infarction;PCI,percutaneous coronary intervention; CABG,coronary artery bypass grafting; TIA, transient ischemic attack; SEE, systemic embolic even; NYHA, New York Heart Association; LV, left ventricle; LVEF, Left ventricular ejection fraction; ACE, Angiotensin-converting enzyme; ARB,angiotensin receptor blocker; CCB, Calcium-channel blocker. CHADS2 score (1 point for congestive heart failure, hypertension, age = 75 years, diabetes, and 2 points for history of stroke or TIA), which is a validated predictor of thromboembolic risk*.

* *Means that there are respective data*.

In order to reduce potential confounders, 29 studies performed statistical models to adjust for clinical variables, which were mainly coming from age, gender, comorbidities, and medications (Table 1). There were 11 studies performed a propensity score-matched cohort analysis to balance baseline characteristics among patients with or not with digoxin therapy, while 25 studies used non-propensity score matched models for statistical adjustment, including Cox regression model, Cox proportional hazards model, and logistic regression model. Only 14 studies provided data of unadjusted HRs for all-cause mortality associated with digoxin use in AF patients.

**Figure 2:**
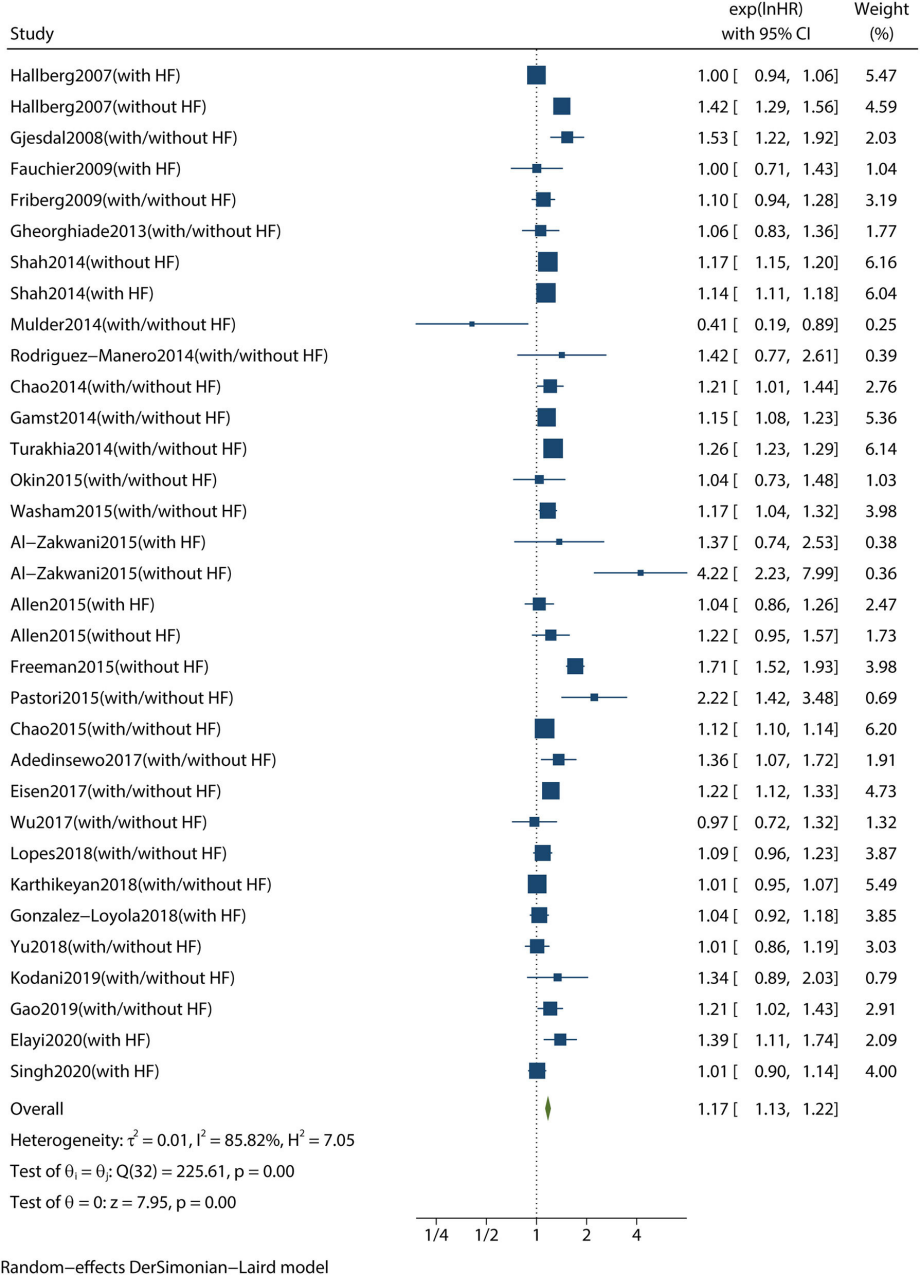
Forest plot showing the effect of digoxin therapy compared with no digoxin therapy on all-cause mortality in patients with AF, regardless of concomitant HF. AF, atrial fibrillation; HF, heart failure; HRs, hazard ratios; CIs, confidence intervals.

**Figure 3:**
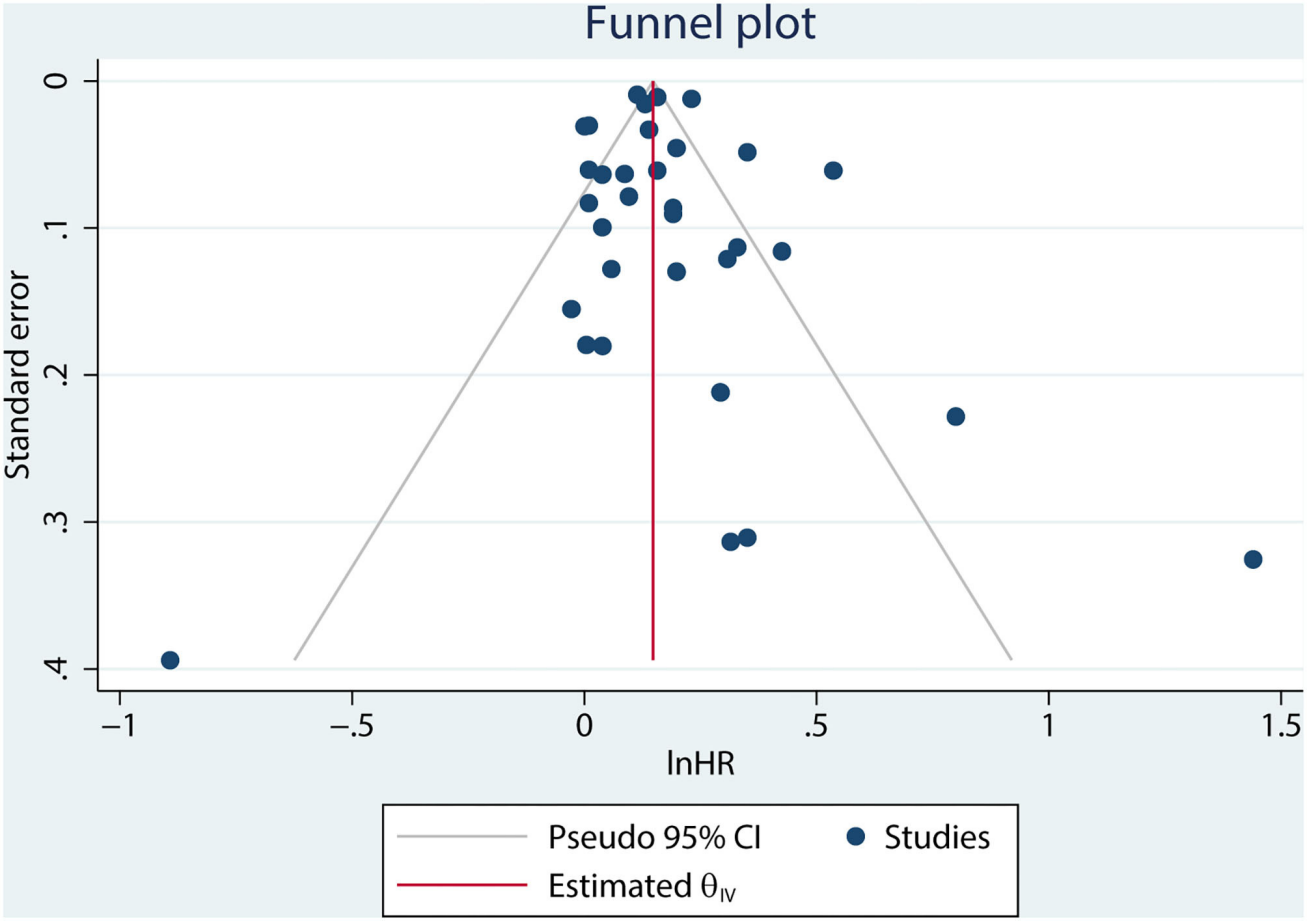
Funnel plot of publications included in the meta-analysis.

A combined analysis of adjusted HRs for all-cause mortality for all patients with AF irrespective of HF showed that digoxin was associated with a significant increase in all-cause mortality (HR 1.17, 95% CI 1.13–1.22, *P* < 0.001, Figure 2). According to Begg's-test (*P* = 0.105), Egger's-test (*P* = 0.221), and visual inspection of the funnel plot (Figure 3), there was no publication bias.

**Figure 4:**
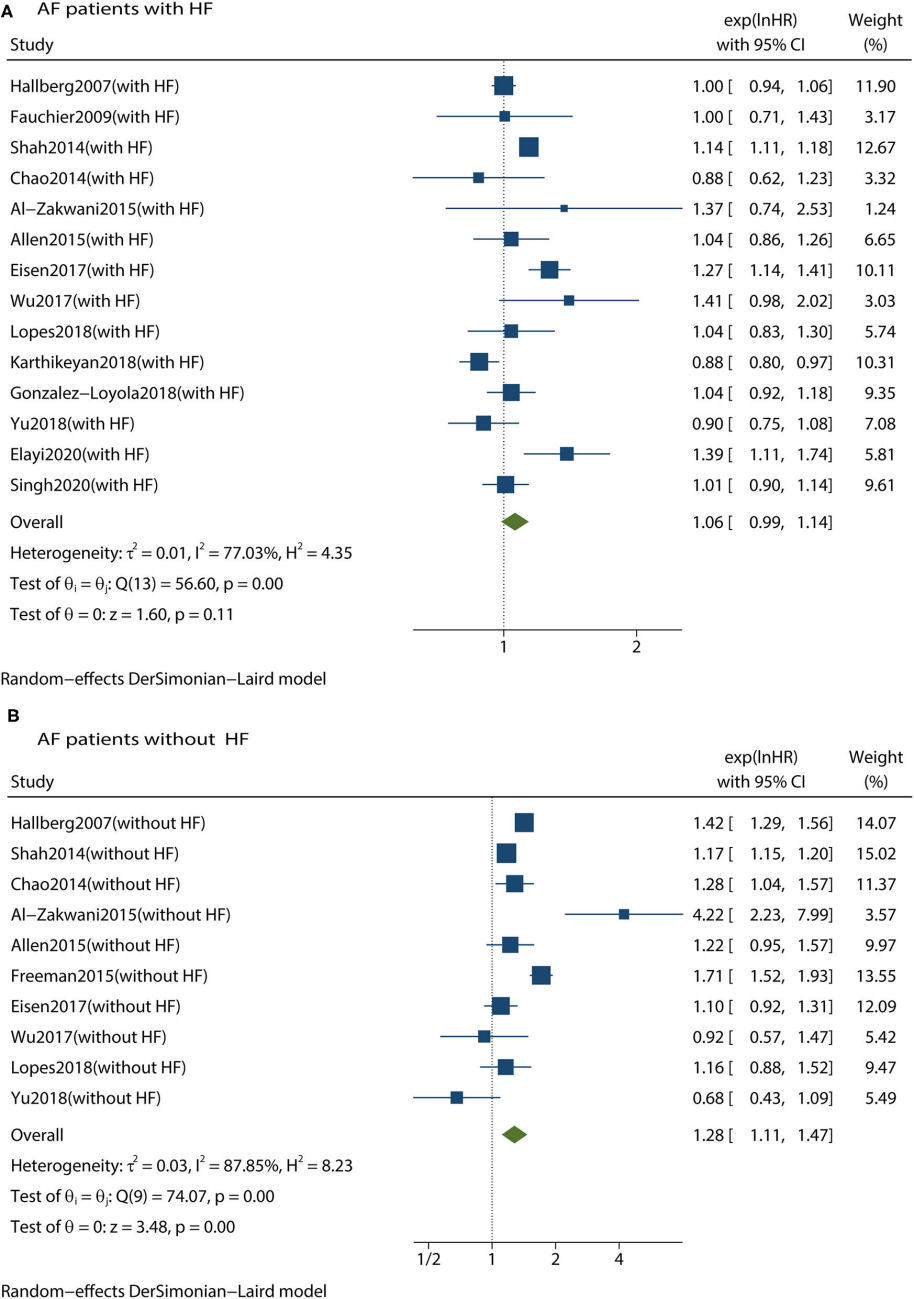
Forest plot showing the effects of digoxin therapy compared with no digoxin therapy on all-cause mortality in AF patients with HF **(A)** or in AF patients without HF **(B)**. AF, atrial fibrillation; HF, heart failure; HRs, hazard ratios; CIs, confidence intervals.

We conducted subgroup analyses according to age, digoxin exposure, study design, statistical methods, follow-up duration, and patients who not receiving β-blockers. We found that digoxin use was still associated with an increased risk of all-cause mortality in patients with AF with or without concomitant HF. Therefore, these subgroups did not have any influence on the primary outcome. We then conducted subgroup analyses to investigate whether the risk of mortality was affected by cardiac function status. In patients with AF without HF, digoxin was associated with a significant increase in all-cause mortality (HR 1.28, 95% CI 1.11–1.47, *P* < 0.001, Figure 4). Meanwhile, in patients with both AF and HF, there was no significant increase in all-cause mortality (HR 1.06, 95% CI 0.99–1.14, *P* = 0.110, Figure 4). These subgroup analyses are presented in Table 2.

**Table 2:** Subgroup analyses of the association between digoxin treatment and all-cause mortality.

**Subgroup**	**Studies**	**Participants**	**HR**	**95% CI**	***P***
**Age**					
≥80 years	3	88,022^a^	1.15	1.12–1.18	<0.001
≥75 years	7	140,652^a^	1.13	1.07–1.19	<0.001
<75 years	20	443,375^a^	1.20	1.13–1.29	<0.001
**Heart function**					
With HF	14	79,824^b^	1.06	0.99–1.14	0.110
Without HF	10	119,538^c^	1.28	1.11–1.47	<0.001
**Digoxin exposure**					
Baseline use	24	550,873^a^	1.17	1.12–1.22	<0.001
Initiate use during follow-up	5	23,372^a^	1.35	1.03–1.78	0.030
**Study design**					
*Post-hoc* analyses of RCTs	8	65,179^a^	1.18	1.07–1.31	<0.001
Observational cohort studies	21	53,152^a^	1.17	1.12–1.22	<0.001
**Statistical methods**					
Unadjusted HR	14	131,880^a^	1.42	1.27–1.59	<0.001
NPSM model (adjusted HR)	25	504,155^a^	1.17	1.11–1.23	<0.001
PSM model (adjusted HR)	11	236,580^a^	1.18	1.12–1.23	<0.001
**Follow-up duration**					
≤ 1 year	6	193,927^a^	1.24	1.09–1.41	<0.001
≥2 years	23	402,773^a^	1.16	1.11–1.20	<0.001
**Patients not receiving** **β-blockers**	5	230,204^a^	1.15	1.04–1.27	< .001

*CI, confidence interval; HR, hazard ratio; HF, heart failure; RCT, randomized controlled trial; PSM, propensity score-matched; NPSM, non-propensity score-matched*.

a *Patients with AF regardless of concomitant HF*.

b *Patients with AF with HF*.

c *Patients with AF without HF*.

We performed additional analyses of the association between digoxin treatment and other clinical outcomes. In all patients with AF regardless of concomitant HF, digoxin use had no significant association with all-cause hospitalization (HR 1.13, 95% CI 0.92–1.39, *P* = 0.230), CV hospitalization (HR 1.05, 95% CI 0.94–1.17, *P* = 0.410), or HF hospitalization (HR 0.99, 95% CI 0.82–1.19, *P* = 0.880). In regard to serious adverse events, our analyses showed no significant increase in SEE /stroke (HR 1.05, 95% CI 0.88–1.25, *P* = 0.590), no significant association with MI (HR 0.96, 95% CI 0.83–1.13, *P* = 0.650), and no significant increase in non-CV mortality (HR 1.08, 95% CI 0.81–1.45, *P* = 0.590) with digoxin. However, there was a significant increase in CV mortality (HR 1.27, 95% CI 1.08–1.50, *P* < 0.001) and SCD (HR 1.40, 95% CI 1.23–1.60, *P* < 0.001) in patients receiving digoxin therapy. Of note, there was no further information about the presence or absence of HF; thus, we could not further analyze the effects of concomitant HF on the above observations. These additional analyses are presented in Table 3.

**Table 3:** Additional analyses of the association between digoxin treatment and other clinical outcomes.

**Outcomes**	**Studies**	**Participants**	**HR**	**95% CI**	***P***
**Hospitalization**					
All-cause hospitalization	7	53,933^a^	1.13	0.92–1.39	0.230
CV hospitalization	4	22,075^a^	1.05	0.94–1.17	0.410
HF hospitalization	4	42,112^a^	0.99	0.82–1.19	0.880
**Serious adverse events**					
SSE/Stroke	6	49,336^a^	1.05	0.88–1.25	0.590
MI	3	36,618^a^	0.96	0.83–1.13	0.650
CV mortality	10	63,975^a^	1.27	1.08–1.50	<0.001
Non-CV mortality	6	49,760^a^	1.08	0.81–1.45	0.590
SCD	5	48,948^a^	1.40	1.23–1.60	<0.001

*CV, cardiovascular; CI, confidence interval; HR, hazard ratio; HF, heart failure; MI, myocardial infarction; SSE, systemic embolic event; SCD, sudden cardiac death*.

a *Patients with AF regardless of concomitant HF*.

## Discussion

This meta-analysis pooled data comparing the effects of digoxin therapy with the effects of no digoxin therapy or other heart rate-controlling drugs in patients with AF. The analysis confirmed that digoxin use is associated with an increased risk of all-cause mortality in patients with AF, regardless of concomitant HF. The risk of all-cause mortality was 17% higher in patients with AF using digoxin compared with those not using digoxin. In the subgroup of patients with AF without HF, the risk of all-cause mortality was 28% higher in patients using digoxin compared with those not using digoxin. Interestingly, in a subgroup of patients with AF with HF, digoxin had no significant association with all-cause mortality. Digoxin was also associated with an increased risk of SCD and CV mortality. Digoxin did not reduce readmission for AF, regardless of concomitant HF.

We confirmed that digoxin use is associated with an increased risk of all-cause mortality in patients with AF, which is similar to previous meta-analyses (11–13, 43). The underlying mechanisms of how digoxin may increase mortality in AF are not yet fully understood; however, several mechanisms may be involved. First, digoxin has potential cardiotoxicity. Cardiac glycosides can cause CV damage by modulating the sodium–potassium ATPase, which is associated with reactive oxygen species production, cardiac remodeling, and arrhythmia (44). Second, digoxin can exacerbate platelet activation in patients with AF, which is associated with an increased incidence of CV disease (45). Pastori et al. (46) found a significant *in-vivo* correlation between serum digoxin concentration and platelet activation. Specifically, a supratherapeutic digoxin concentration increased platelet aggregation. Third, digoxin is cleared by the kidney and has a narrow therapeutic window. Digoxin interacts with many other drugs, leading to increased serum digoxin concentration and increasing the risk of arrhythmia (47). Many older and sicker patients have renal insufficiency and may concomitantly use drugs that could increase serum digoxin concentration. Elevated serum digoxin may lead to side effects and toxicity.

When all included patients with AF were stratified by cardiac function status at baseline, we found that in patients with AF without HF, digoxin was still associated with an increased risk of all-cause mortality, but there was no evidence of an increase in all-cause mortality in patients with AF with HF, which is different from several previous meta-analyses (11, 43, 48–50). In our study, we included a greater number of studies compared with previous meta-analyses, which provided data on digoxin treatment in patients with both AF and HF. The majority of these studies reported no significant association between digoxin and mortality in patients both with AF and HF (10, 15, 23, 25, 31, 35, 37–39, 41). Even the most recent RCT, which compared the clinical effects of low-dose digoxin with bisoprolol in patients with AF with symptoms of HF, found better symptom control with digoxin for both AF- and HF-related symptoms, which is consistent with a lower N-terminal pro-B-type natriuretic peptide concentration and fewer adverse events (51). Digoxin has positive inotropic effects, negative chronotropic effects, and anti-adrenergic effects (7). These effects are thought to be beneficial in patients with AF and HF, which may be a reason for the inconsistent results in patients with AF without HF. However, According to AFFIRM study (52), β-blockers are the most effective drug for heart rate control, digoxin is usually a second-line option or combination with β-blockers, maybe it is not the neutral mortality effect of digoxin use in patients with AF with HF, but other heart rate control agents like β-blockers which could improve survival. Therefore, we conducted subgroup analyses in patients not receiving β-blockers, there were five studies reported data of digoxin alone VS no other rate control treatment in patients with AF with or without HF, in which all the HRs were adjusted by non-propensity score matched models, also indicating increased mortality associated with digoxin therapy.

It should be noted that our meta-analysis was based on data from observational studies or *post-hoc* analyses of RCTs. Because most RCTs limited follow-up of days to weeks, and did not evaluate long-term mortality or hospitalization. Recently, Sethi et al. (16) performed a meta-analysis of digoxin for heart rate control in patients with AF, which was based on data from RCTs. They indicated that the clinical effects of digoxin on all-cause mortality and serious adverse events are unclear based on current evidence, because no trials included in this meta-analysis reported long term follow-up data. At present, more information is required from large-sample observational studies to help us learn about the long-term effects of digoxin.

As we know, digoxin is more commonly used in patients who have a greater comorbid burden and who require additional heart rate-controlling therapy; in other words, patients treated with digoxin are generally sicker than those not requiring digoxin, thus leading to selection bias. Therefore, digoxin initiation during follow-up might avoid potential selection bias originating from baseline use of digoxin. When we pooled studies reporting digoxin initiation during follow-up in patients with AF, digoxin was still associated with an increased risk of all-cause mortality.

In order to reduce potential confounders, some studies usually used Cox regression or logistic regression model to adjust for clinical variables in observational studies, some studies performed a propensity score-matched cohort to select digoxin and no-digoxin treatment groups that were well-balanced on various patient-related baseline characteristics. In our subgroup analyses, the combined analysis of unadjusted HRs for mortality in patients with AF suggested the risk of all-cause mortality was 42% higher in patients using digoxin compared with those not. However, after adjustment for baseline differences, the risk of all-cause mortality was lower in subgroups which HRs were adjusted by statistical models. And the results of these subgroup analyses still showed that treatment with digoxin in patients with AF is associated with an increased risk of all-cause mortality.

Ziff et al. (53) suggested that digoxin use is associated with a reduction in hospital admissions. Singh et al. (15) also indicated that digoxin is associated with a lower risk of HF readmission. However, in our study, we found no evidence of a reduction in readmission in overall patients with AF. With regard to serious adverse events during follow-up, we found that digoxin use has no significant association with SEE/stroke, MI, and non-CV mortality, but the risks of SCD and CV mortality were 40 and 27%, respectively. These rates were higher in patients with AF using digoxin compared with those not using digoxin. SCD events occupy a proportion of CV mortality events. Eisen et al. (36) examined the association between baseline features and SCD in patients with AF. They found that digoxin use was a significant predictor of SCD in patients with AF. This increase in SCD might be attributed to arrhythmic death based on the mechanism of action of digoxin (10).This could be one underlying explanation for the digoxin-associated increase in the risk of SCD.

## Study Limitations

There are several limitations of our study that should be noted. First, our data were mainly based on observational studies, and we observed substantial heterogeneity in most analyses, which may not be evident in meta-analyses of RCTs (53). And statistical adjustment of observational data and propensity-score matching cannot replace randomized allocation. Second, we considered that the risk of secondary outcomes in patients with AF was associated with digoxin therapy, which may be different when patients with AF are stratified by cardiac function status. Third, we could not conduct analysis of digoxin concentration and relevant risk of mortality, because digoxin concentration was not available in most of the included studies. Given the above limitations, we hope that more future studies assessing the clinical effects of digoxin in patients with AF could stratify patients according to cardiac function status and provide data of serum digoxin concentration.

## Conclusion

Digoxin use is associated with an increased risk of all-cause mortality in patients with AF, especially those without concomitant HF. Digoxin use is also associated with an increased risk of SCD and CV mortality, and digoxin does not seem to reduce readmission for AF, regardless of concomitant HF. But digoxin may have a neutral effect on all-cause mortality in patients with AF with concomitant HF. Thus, digoxin might be an additional choice for heart rate control in patients with both AF and HF, particularly in patients who are unable to tolerate β-blockers or do not achieve their target heart rate. However, we suggest that digoxin should be used cautiously with appropriate concentration monitoring to avoid toxicity.

## Data Availability Statement

The original contributions presented in the study are included in the article/Supplementary Material, further inquiries can be directed to the corresponding author.

## Author Contributions

XW: conceptualization, literature searches, data extraction, formal analysis, and writing-original draft. YL and DX: literature searches and data extraction. KZ: review and editing. All authors contributed to the article and approved it for publication.

## Conflict of Interest

The authors declare that the research was conducted in the absence of any commercial or financial relationships that could be construed as a potential conflict of interest.

## Publisher's Note

All claims expressed in this article are solely those of the authors and do not necessarily represent those of their affiliated organizations, or those of the publisher, the editors and the reviewers. Any product that may be evaluated in this article, or claim that may be made by its manufacturer, is not guaranteed or endorsed by the publisher.
