# Supplementary material for: Effect of Digoxin Therapy on Mortality in Patients With Atrial Fibrillation: An Updated Meta-Analysis
Source: Front Cardiovasc Med. 2021 Oct 1;8:731135. doi: 10.3389/fcvm.2021.731135 (PMC8517124; doi:10.3389/fcvm.2021.731135)
Supplement: Supplementary file 1 [file Table_1.DOC]

**Search strategy in PubMed**

| Search | Query |
| --- | --- |
| #1 | "atrial fibrillation"[MeSH Terms] |
| #2 | "Atrial"[Title/Abstract] OR "auricular"[Title/Abstract] |
| #3 | "fibrillation*"[Title/Abstract] OR "tachycardia*"[Title/Abstract] OR "tachyarrhythmia"[Title/Abstract] OR "arrhythmia*"[Title/Abstract] OR "flutter*"[Title/Abstract] |
| #4 | #2 AND #3 |
| #5 | "AF"[Title/Abstract] |
| #6 | #1 OR #4 OR #5 |
| #7 | "digoxin"[MeSH Terms] |
| #8 | "digoxin"[Title/Abstract] OR "digitalis"[Title/Abstract] OR “digitoxin”[Title/Abstract] |
| #9 | #7 OR #8 |
| #10 | #9 AND #6 |
| #11 | cohort studies[Mesh:NoExp] OR longitudinal studies[Mesh:NoExp] OR follow-up studies[Mesh:NoExp] OR prospective studies[Mesh:NoExp] OR retrospective studies[Mesh:NoExp] OR cohort[Title/Abstract] OR longitudinal[Title/Abstract] OR prospective[Title/Abstract] OR retrospective[Title/Abstract] |
| #12 | #10 and #11 |
