# Supplementary material for: Effect of Digoxin Therapy on Mortality in Patients With Atrial Fibrillation: An Updated Meta-Analysis
Source: Front Cardiovasc Med. 2021 Oct 1;8:731135. doi: 10.3389/fcvm.2021.731135 (PMC8517124; doi:10.3389/fcvm.2021.731135)
Supplement: Supplementary file 2 [file Table_2.DOCX]

Search strategy in Cochrane Library

| Search | Query |
| --- | --- |
| #1 | MeSH descriptor: [atrial fibrillation] explode all trees |
| #2 | (Atrial):ti, ab, kw OR (auricular):ti, ab, kw |
| #3 | (fibrillation*):ti, ab, kw OR (tachycardia*):ti, ab, kw OR (tachyarrhythmia):ti, ab, kw OR (arrhythmia*) OR (flutter*):ti, ab, kw |
| #4 | #2 AND #3 |
| #5 | (AF):ti, ab, kw |
| #6 | #1 OR #4 OR #5 |
| #7 | MeSH descriptor:[digoxin] explode all trees |
| #8 | (digoxin) :ti, ab, kw OR (digitalis) :ti, ab, kw or (digitoxin) :ti, ab, kw |
| #9 | #7 OR #8 |
| #10 | #9 AND #6 |
| #11 | MeSH descriptor:[cohort studies] OR MeSH descriptor:[longitudinal studies] OR MeSH descriptor:[follow-up studies] OR MeSH descriptor:[prospective studies] OR MeSH descriptor:[retrospective studies] OR (cohort) :ti, ab, kw OR (longitudinal) :ti, ab, kw OR (prospective) :ti, ab, kw OR (retrospective) :ti, ab, kw |
| #12 | #10 and #11 |
