# Supplementary material for: Effect of Digoxin Therapy on Mortality in Patients With Atrial Fibrillation: An Updated Meta-Analysis
Source: Front Cardiovasc Med. 2021 Oct 1;8:731135. doi: 10.3389/fcvm.2021.731135 (PMC8517124; doi:10.3389/fcvm.2021.731135)
Supplement: Supplementary file 3 [file Table_3.DOCX]

Search strategy in Embase

| Search | Query |
| --- | --- |
| #1 | ‘atrial fibrillation’/exp |
| #2 | atrial:ab,ti OR auricular:ab,ti |
| #3 | fibrillation*:ab,ti OR tachycardia*:ab,ti OR tachyarrhythmia:ab,ti OR "arrhythmia*:ab,ti OR flutter*:ab,ti |
| #4 | #2 AND #3 |
| #5 | AF:ab,ti |
| #6 | #1 OR #4 OR #5 |
| #7 | ‘digoxin’/exp |
| #8 | digoxin:ab,ti OR digitalis:ab,ti OR digitoxin:ab,ti |
| #9 | #7 OR #8 |
| #10 | #9 AND #6 |
| #11 | ‘cohort studies’/ OR ‘longitudinal studies’/ OR ‘follow-up studies’/ OR ‘prospective studies’/ OR ‘retrospective studies’/ OR cohort:ab,ti OR longitudinal:ab,ti OR prospective:ab,ti OR retrospective:ab,ti |
| #12 | #10 and #11 |
